# Simultaneous recovery of high-purity Cu and poly(vinyl chloride) from waste wire harness via swelling followed by ball milling

**DOI:** 10.1038/s41598-020-67795-9

**Published:** 2020-07-01

**Authors:** Harendra Kumar, Shogo Kumagai, Tomohito Kameda, Yuko Saito, Toshiaki Yoshioka

**Affiliations:** Graduate School of Environmental Studies, Tohoku University, 6-6-07 Aoba, Aramaki-Aza, Aoba-ku, Sendai, Miyagi 980-8579 Japan

**Keywords:** Sustainability, Environmental chemistry

## Abstract

Poly(vinyl chloride) (PVC) swelling coupled with ball milling was employed for the simultaneous recovery of high-purity Cu and PVC from waste wire harness under ambient conditions. The experimentally determined performances of 15 organic solvents for PVC swelling and phthalate plasticiser extraction were compared with those predicted considering Hansen solubility parameters. As a result, *n*-butyl acetate and acetone were identified as the two best solvents for adequate PVC swelling without PVC dissolution and almost complete plasticiser extraction within 60 min. The swelling was concluded to contribute to the control of phthalate plasticisers, the use of which in wire harness has recently been limited by the Restriction of Hazardous Substances (RoHS) directive. Cables swollen with *n*-butyl acetate or acetone were subjected to dry ball milling for ~ 60 min to completely separate PVC and Cu and achieve the quantitative recovery of these components from 20-cm-long cables. Thus, this work unveils the high potential of recycling the otherwise non-recyclable long and non-uniform waste wire harness cables and is expected to impact the related (e.g., automotive, electrical, and electronics) industries, contributing to the establishment of a more sustainable society.

## Introduction

Electric cables are indispensable for electricity/information transmission and contain poly(vinyl chloride) (PVC) and Cu as major constituents accounting for almost 16 and 42%, respectively, of the total material consumption in cable production^1^. Cu exhibits good electrical conductivity, and the corresponding applications have a 58% share of the global Cu demand (estimated at 25 and 28 million tonnes in 2015 and 2019, respectively), with 30% of this demand fulfilled by recycling^2^. PVC is well suited for coating Cu wire strands and is commonly made more flexible through the incorporation of plasticisers such as diisononyl phthalate (DINP) and di-2-ethylhexyl phthalate at loadings of 18–40 wt%^3^. Wire harness is the major component of the cable waste stream, being hard to process because of the low thickness and non-uniformity of the cable assembly, which usually consists of Cu strands coated with a single layer of PVC^4^.

The wire harness assembly, used to conduct electricity to the components of vehicles and electrical/electronic appliances, is abundantly present in waste electrical/electronic equipment and end-of-life vehicles (ELVs), the production of which is steadily increasing (> 40 million automobiles are discarded per year^5^). On average, a vehicle contains ~ 30 kg of Cu, with wire harness accounting for 50% of this value^6^. On the other hand, wire harness accounts for only 2% of the 50 million tonnes of e-waste generated annually^7^. As Cu has a higher economic value than PVC, the recycling of wire harness in some places of India^8^, China^9^, and Africa^10^ is performed by open burning to recover Cu. As PVC contains ~ 57% chlorine by weight and thus emits undesired HCl^11^ (and possibly dioxins^12^) upon burning, this recycling method poses a threat to the environment. Moreover, the pigments and stabilisers used in cable PVC coating may contain hazardous Pb and Cd compounds, and the landfilling of burnt PVC residues may therefore result in soil and water contamination^8^.

In view of the above, much effort has been directed at the development of a safe and competitive method of PVC and Cu recycling. Koyanaka et al*.*^13^ used mechanical grinding and crushing as an environmental friendly technique of recycling thin cables. However, this method suffered from low Cu recovery, i.e., much Cu was discarded with PVC residues. To mitigate this problem, chemical/bioleaching^14^ was additionally employed to recover Cu from cable residue, and a Cu recovery of > 90% was achieved using *Acidithiobacillus ferrooxidans* in the case of bioleaching. However, this technique features excessive solvent use and thus raises pollution concerns. Chloride volatilisation^15^ via treatment with an appropriate gas mixture (HCl, N_2_, air) resulted in a > 80% Cu recovery (as CuCl) at 900 °C. Despite being effective for cable residues, this method is not environmentally friendly because of its high thermal energy consumption. The ability of PVC to be dissolved with organic solvents has been employed in the recycling of PVC-coated polyethylene terephthalate fibre^16^. Moreover, Solvay (VINILOOP) recycled waste PVC cables^17^ by dissolution of the PVC coating to recover Cu, with subsequent PVC recovery achieved by precipitation. The advantages of this technique are the avoidance of pre-treatments such sorting/cutting and the recovery of high-grade Cu, while the drawbacks are the multistep processes for plasticiser recovery and solvent regeneration.

Thin cable recycling has also been attempted by extraction-induced PVC embrittlement followed by crushing^18,19^. In this case, 1-cm-long cables were subjected to quantitative plasticiser extraction and then ball milled to achieve full recovery of ~ 100% pure Cu, although the recycling of longer or non-uniform cables was problematic. To retain existing PVC properties, Xu et al*.*^20,21^ developed a method based on swelling and mechanical agitation. Specifically, swelling created a gap between Cu and the PVC coating and thus facilitated their separation, obviating the need for PVC dissolution. More importantly, the above authors realised the recovery of plasticiser-free PVC using organic solvents. However, only cables less than 5 cm long could be recycled by this method.

The advantages and disadvantages of the abovementioned techniques highlight the need for a process allowing one to recycle long, thin, and non-uniform cables and achieve high recoveries of pure Cu. Moreover, the controlled (or complete) extraction of plasticisers must be taken into account, as phthalates are categorised as hazardous substances according to the Restriction of Hazardous Substances (RoHS) directive. Fully plasticiser-free PVC can be incorporated into flooring and drain/sewer pipes^22^ by blending^23^, and the retrieved plasticisers can be re-used in the PVC industry.

**Figure 1:**
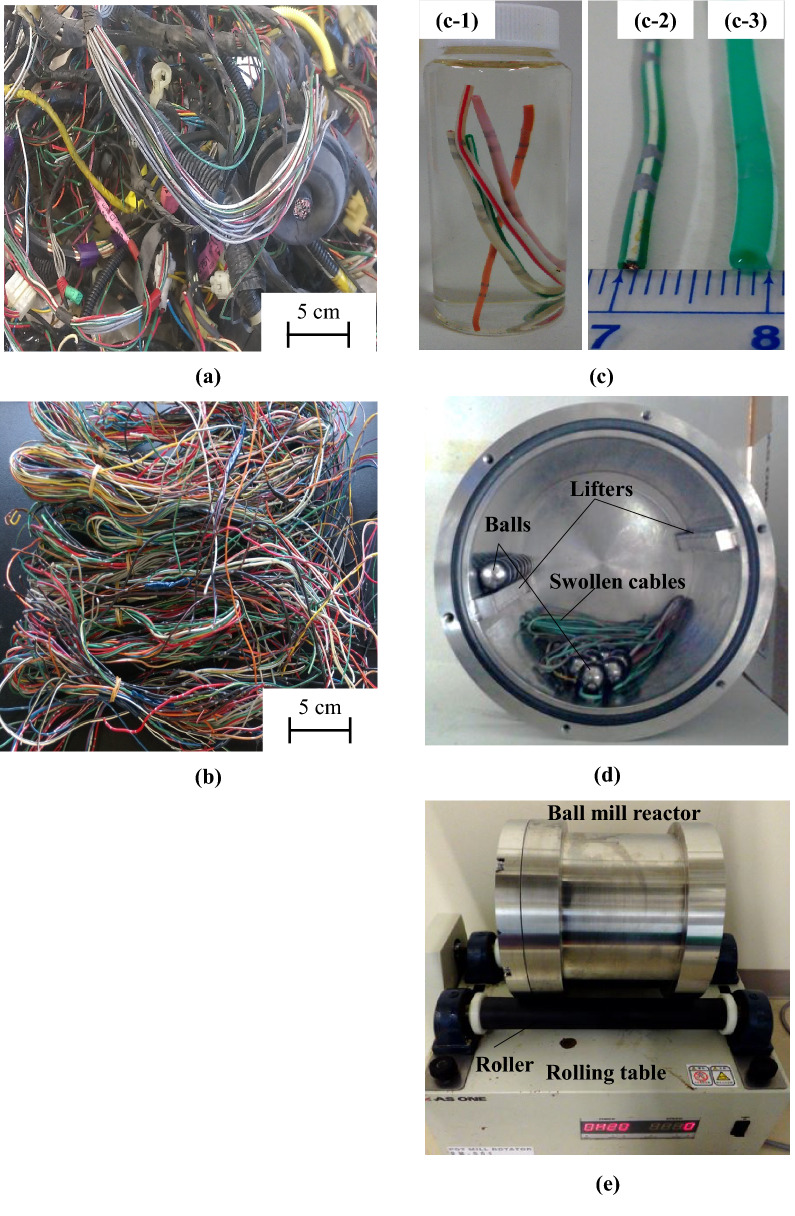
(**a**) Wire harness collected from waste ELVs, (**b**) cables with various diameters and textures after the sorting out of connectors, bands, and covers, schematics of wire harness recycling: (**c**) anticipated cable swelling [(**c-1**) cable immersed into organic solvent, (**c-2**) original cable, (**c-3**) swollen cable)], (**d**) feeding of swollen cables into the ball mill reactor with balls (inside view of the ball mill reactor), (**e**) swollen cable separation by ball milling (complete setup).

Herein, we show that non-uniform, long, and thin cables can be recycled in an environmentally friendly way by adequate swelling of their PVC coating followed by ball milling to afford Cu and solid PVC (Fig. 1). The PVC swelling and extraction relationship was developed within the framework of the polymer solubility concept according to Hansen solubility parameters (HSPs), and the effects of milling conditions on the recovery of PVC and Cu were investigated in detail.

## Results and discussion

### Characterisation of wire harness samples

Waste wire harness assemblies were collected from ELVs (Fig. 1a) and contained cables of various diameters/textures, connectors, and banding bands. The connectors and banding bands were manually removed, and the cables were retained (Fig. 1b). Cables with diameters of 1.0, 1.2, and 2.0 mm had shares of 15, 70, and 15 wt%, respectively (Supplementary 1), and were all coated with PVC according to the results of elemental analysis (Supplementary Table S1). The quantitation of Cu, PVC resin, plasticiser, and tetrahydrofuran (THF)-insoluble components and plasticiser identification were conducted as described in Supplementaries 2 and 3, with the obtained results summarised in Table 1. The average contents of Cu and PVC were estimated at 71.1 and 28.9 wt%, respectively, taking into consideration the above cable distribution (Supplementary Fig. S1). ^1^H NMR analysis revealed that samples contained DINP as a plasticiser at a content of 21.4 wt% (Supplementaries 3 and 4), and insoluble compounds accounted for 6.7 wt% of the PVC coating. Thus, the present work quantitatively evaluated the composition of the heterogeneous waste wire harness assembly.

**Table 1:** Contents of Cu and PVC as well as PVC coating formulations for different cable sizes and a selected group of cable samples.

Cable diameter (mm)	Thickness of PVC coating (mm)	Composition (wt%)						
		Cu	PVC coating	Avg. Cu^a^	Avg. PVC coating^a^	Composition of PVC coating		
						PVC resin	DINP	Insoluble components^b^
1.0	0.12	68.0	32.0	71.1	28.9	71.9	21.4	6.7
1.2	0.24	70.6	29.4					
2.0	0.25	79.1	20.9					

^a^Calculated by considering the cable distribution (70 wt% of 1.2-mm-diameter cables and 15 wt% of 1.0- and 2.0-mm-diameter cables.

^b^CaCO_3_, TiO_2_, flame retardant such as Sb_2_O_3_, and cross-linked PVC.

### Swelling of PVC coating and DINP extraction by organic solvents

**Figure 2:**
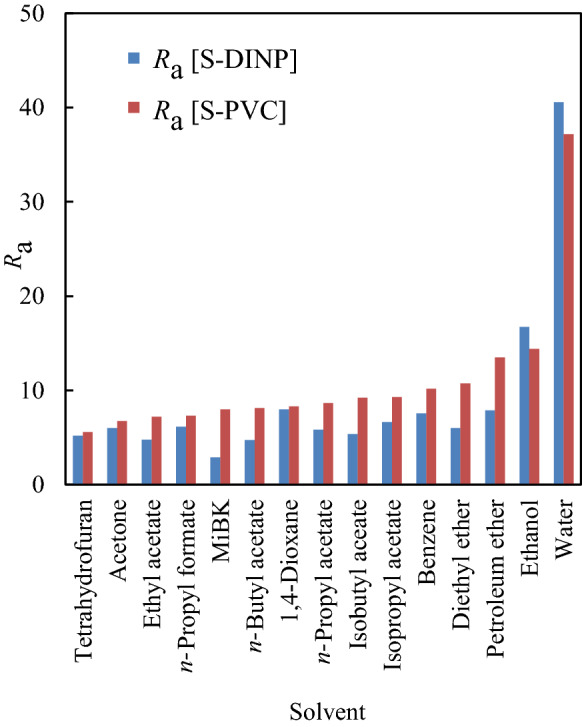
HSP distances between solvents (S) and DINP (*R*_a_ [S-DINP]) and between solvents and PVC (*R*_a_ [S-PVC]).

Swelling allows the PVC coating to be easily separated by crushing in a ball mill reactor. Herein, 15 solvents covering a broad range of solvent-PVC HSP interaction distances (*R*_a_ [S-PVC], calculated by Eq. (1) and summarised in Fig. 2) were selected, with details of each HSPs summarised in Supplementary Table S5-1. Table 2 summarises the abilities of organic solvents to swell PVC at ambient temperature, showing that according to this ability, the solvents can be divided into inadequate (i.e., benzene, diethyl ether, petroleum ether, ethanol, and water, *R*_swel_ ≤ 2), moderate (ethyl acetate, isobutyl acetate, and isopropyl acetate, 2 ≤ *R*_swel_ ≤ 3), adequate (acetone, *n*-propyl formate, *n*-butyl acetate, 1,4-dioxane, *n*-propyl acetate, 3 ≤ *R*_swel_ ≤ 4 ), partial dissolution (4-methyl-2-pentanone (MiBK), 4 ≤ *R*_swel_), and complete dissolution (THF, dissolution) groups. THF and MiBK, which completely and partially dissolved PVC coatings, respectively, were thus considered unsuitable for swelling. Although the order of *R*_swel_ did not completely match that of *R*_a_ [S-PVC], solvents suitable for adequate PVC swelling were concluded to have *R*_a_ [S-PVC] = 6–9. A detailed discussion regarding the effects of HSP on PVC swelling and the corresponding mechanism are provided in Supplementary 5. Notably, shaking did not substantially impact swelling behaviour, i.e., simple submersion was sufficient for obtaining swollen cables.

**Table 2:** Ability of organic solvents to swell and shrink PVC coatings.

Solvent	Volumetric swelling ratio (*R*_swel_) (–)				
	Without shaking				With shaking
	20 min (18 °C)	40 min (18 °C)	60 min (18 °C)	80 min (18 °C)	60 min (20 °C)
THF	Dissolution				
Acetone	1.9 ± 0.1^a^	2.4 ± 0.0	2.9 ± 0.0	3.0 ± 0.0	3.0 ± 0.1
Ethyl acetate	1.7 ± 0.1	1.9 ± 0.1	2.1 ± 0.1	2.5 ± 0.0	2.4 ± 0.0
*n*-Propyl formate	2.3 ± 0.1	2.8 ± 0.0	2.9 ± 0.1	3.0 ± 0.0	3.1 ± 0.1
MiBK	3.9 ± 0.0	5.0 ± 0.0	5.2 ± 0.1	5.7 ± 0.1	5.2 ± 0.0
*n*-Butyl acetate	2.4 ± 0.0	3.1 ± 0.0	3.4 ± 0.1	3.5 ± 0.1	3.4 ± 0.1
1,4-Dioxane	2.2 ± 0.0	2.9 ± 0.0	3.3 ± 0.0	3.3 ± 0.0	3.3 ± 0.0
*n*-Propyl acetate	2.8 ± 0.0	3.3 ± 0.0	3.3 ± 0.0	3.3 ± 0.1	3.4 ± 0.1
Isobutyl acetate	2.1 ± 0.0	2.3 ± 0.1	2.5 ± 0.1	2.5 ± 0.1	2.6 ± 0.0
Isopropyl acetate	1.9 ± 0.1	2.1 ± 0.0	2.2 ± 0.1	2.2 ± 0.1	2.2 ± 0.1
Benzene	1.1 ± 0.0	1.2 ± 0.0	1.2 ± 0.0	1.3 ± 0.0	1.2 ± 0.1
Diethyl ether	0.9 ± 0.0	0.9 ± 0.0	0.9 ± 0.1	0.9 ± 0.0	0.9 ± 0.0
Petroleum ether	1.0 ± 0.0	1.0 ± 0.0	1.0 ± 0.0	0.9 ± 0.0	0.9 ± 0.1
Ethanol	1.0 ± 0.0	1.0 ± 0.0	1.0 ± 0.0	1.0 ± 0.0	1.0 ± 0.0
Water	1.0 ± 0.0	1.0 ± 0.0	1.0 ± 0.0	1.0 ± 0.0	1.0 ± 0.0

^a^Standard deviation determined from ten results of 10 experiments.

**Figure 3:**
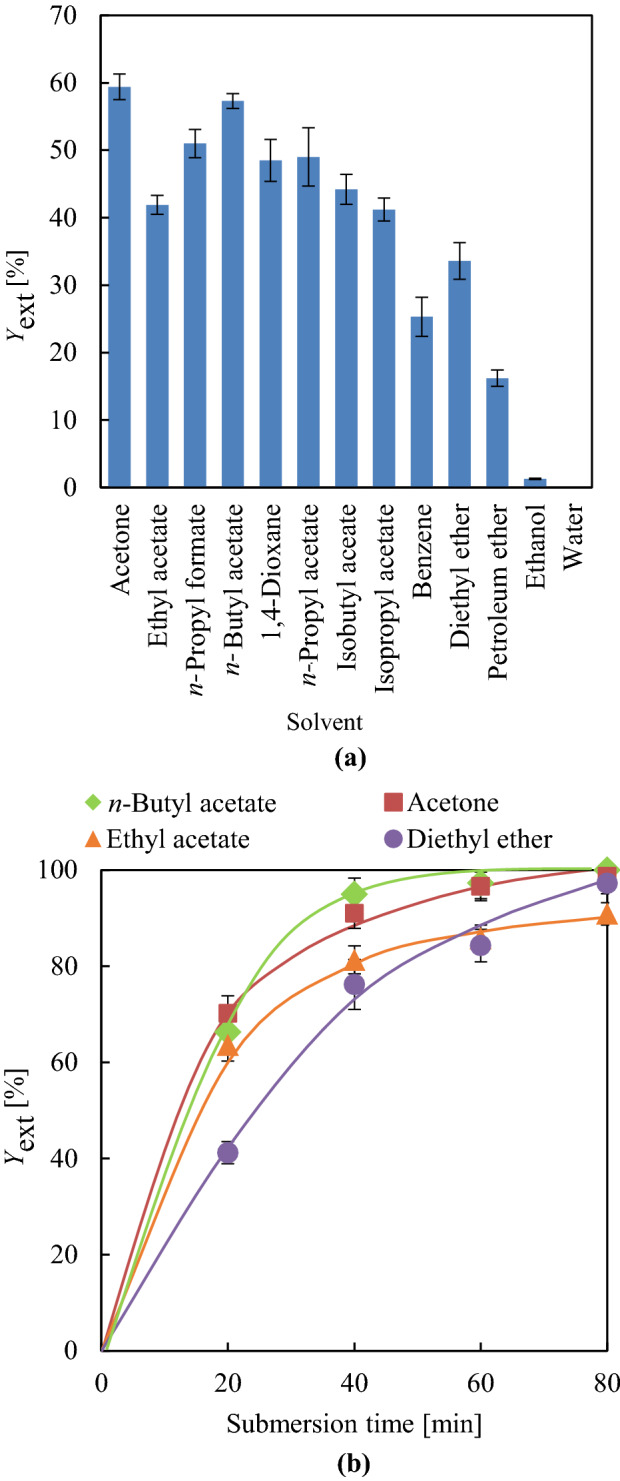
(**a**) DINP *Y*_ext_ [%] at 80 min without shaking and (**b**) time-dependent evolution of *Y*_ext_ with shaking for acetone, *n*-butyl acetate, ethyl acetate, and diethyl ether.

Figure 2 presents HSP interaction distances between each solvent and DINP (*R*_a_ [S-DINP]). The best plasticiser extraction yield (*Y*_ext_) of ~ 60% was observed for shaking-free 80-min extraction with acetone and *n*-butyl acetate (Fig. 3a). Although the order of *R*_swel_ does not perfectly match that of *Y*_ext_ for each solvent, these parameters are strongly mutually correlated, as PVC swelling assists solvent penetration into the PVC matrix. Therefore, the behaviour of *Y*_ext_ cannot be simply explained by that of *R*_a_ [S-DINP]. DINP extraction was substantially accelerated by shaking, with the effects of time on the *Y*_ext_ of diethyl ether, ethyl acetate, *n*-butyl acetate, and acetone shown in Fig. 3b. The related results for other solvents are summarised in Supplementary Table S5-2. The *Y*_ext_ values of each solvent obtained after 20 min under shaking conditions exceeded those obtained after 80 min without shaking, i.e., shaking substantially enhanced DINP extraction efficiency. The maximal DINP extraction efficiencies of 99 and 97% at 80 min were achieved using acetone and *n*-butyl acetate, respectively. Although the *R*_swel_ values of ethyl acetate and diethyl ether were lower than those of acetone and *n*-butyl acetate, the *Y*_ext_ values of the former group (91%) were comparable to those of the latter (97%) at 80 min. Thus, almost complete DINP extraction could be achieved using supportive shaking motion, whereas this motion did not influence PVC swelling.

Thus, solvents adequately swelling PVC without its dissolution were concluded to be appropriate for subsequent dry ball milling, while solvents with high DINP extraction abilities were found to be beneficial for the control of RoHS-regulated phthalate plasticisers. Thus, taking the cost factor into account, acetone, *n*-butyl acetate, and ethyl acetate were selected as inexpensive and safe (i.e., posing low health and environmental hazards)^24^ solvents for further experiments.

### Insights into the mechanism of PVC solvation and plasticiser extraction

**Figure 4:**
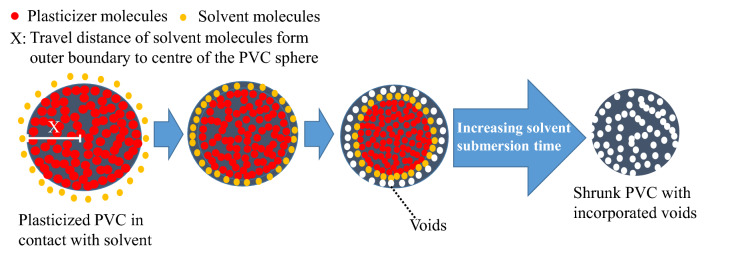
General mechanism of plasticiser extraction without PVC matrix swelling to result in post-extraction shrinkage.

Polymer dissolution is controlled by either the disentanglement of chains or their diffusion at the polymer (solid)-solvent interface^25^. Herein, the dissolution of PVC in THF was controlled by chain diffusion, and no swelling interface was observed before dissolution. At sufficiently high solvent penetration and swelling power, the swollen layer starts to be disentangled, as in the case of MiBK. Furthermore, the PVC coating contained encapsulated DINP, which could be released by swelling or chemical interactions. However, swelling is known to expand the PVC matrix and thus facilitate extraction. Hence, boundary layer resistance appears, particularly when the solvent affinity to PVC is low. Solvent molecules interact with incorporated DINP molecules at the outer boundary layer and initiate extraction to create voids (free surface), which allows for the further diffusion of solvent molecules and their interaction with deeper-lying DINP molecules. This process results in weight loss (shrinkage), as the solvent finally leaves the occupied voids. Figure 4 shows how DINP is extracted from the PVC matrix without swelling, while shrinkage (weight loss) is observed after extraction. Solvents with modest swelling power such as acetone, ethyl acetate (EA), *n*-butyl acetate, *n*-propyl acetate, and 1,4-dioxane featured a slow swelling extent that was maximised at the end of the swelling time, leaving adjacent PVC chains unaffected. However, EA achieved a very limited swelling extent. Notably, only shrinkage was observed in diethyl ether and petroleum ether, i.e., these solvents engaged in favourable chemical interactions with DINP but not with PVC. To validate shrinkage behaviour of PVC by weight loss into diethyl ether, petroleum ether, and ethanol, herein *R*_swel_ tests of fully deplasticized were conducted, as results *R*_swel_ exhibited unity for all three respective solvents after 60-min submersion, indicate that there was no weight loss occurred during swelling tests, while as *R*_swel_ was tested < 1 for plasticised cables. Moreover, the *R*_swel_ values of specimens (see Table 2) fully extracted upon shaking were not different from values of samples extracted by conventional submersion (dipping).

**Figure 5:**
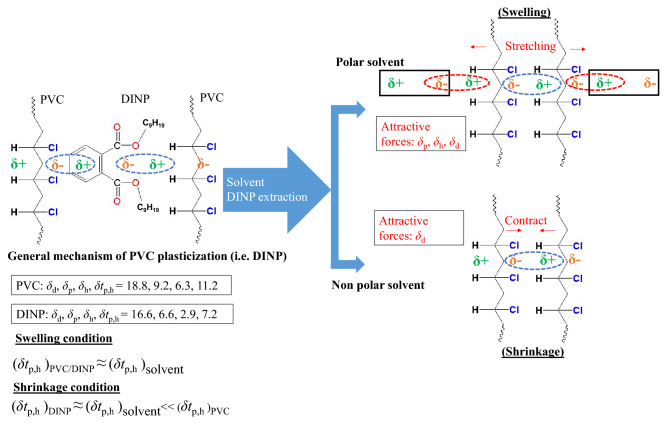
General demonstration of PVC swelling and shrinkage under influence of organic solvents.

Notably, the solvent-PVC HSP interaction distance, *R*_a_ [S-PVC], was positively correlated with the extent of solvation. Polar forces such as those due to *δ*_d_ and *δ*_h_ (as opposed to forces due to *δ*_d_) are highly directional and greatly influence solvation. For example, ethanol exhibited *δ*_d_ = 15.8, which was close to that of THF (16.8); however, THF dissolved PVC (*δ*_d_ = 18.8), while ethanol completely incompetent solvent in solvation of PVC, makes sense that *δ*_p_ and *δ*_h_ substantially influence PVC solvation. Considering *δ*_p_ and *δ*_h_ to describe the order of solvation, we note that solvents (e.g., diethyl ether and petroleum ether) with either low or very high *δ*_p_ and *δ*_h_ [(2.9 ≤ *δ*_p_ ≥ 0) and (5.1 ≤ *δ*_h_ ≥ 0)] induced no significant chemical changes of PVC, which has *δ*_p_ = 9.2 and *δ*_h_ = 6.3. Still, shrinkage (*R*_swel_ < 1) was observed for diethyl ether/petroleum ether because of plasticiser extraction from the PVC matrix. Moreover, while PVC-diethyl ether/petroleum secondary forces are exceptionally weaker for PVC swelling, shrinkage may impart due to fact that induced secondary forces of PVC–PVC attempt to contract chains by occupied free space (i.e., created by leaving plasticiser molecules from PVC matrix). Unlike the above ethers, benzene achieved a certain degree of PVC swelling, which was ascribed to the interaction of the benzene π-electron cloud with PVC molecules. However, this hypothesis is not backed up by evidence. Another viewpoint on polymer solubility^26^ that is difference in free volume between the polymer and solvent liquids, i.e., dissimilarity mainly due to differences of sizes of solvent molecules and polymers molecules (i.e., fashioning of chains), may influence polymer solvation, aside of induced contact energy dissimilarities. Likely, Gilbert^27^ also highlighted the contribution of molecular size and chemical structure to polymer solvation. The *δ*_p_ and *δ*_h_ deviate in small for THF (*δ*_p_ = 5.7, *δ*_h_ = 8), and EA (*δ*_p_ = 5.3, *δ*_h_ = 7.2), wherein THF dissolve PVC, while EA *R*_swel_ exhibited < 3, is very far from PVC dissolution. Although, induced forces are important for discussion but hard to explain individual influence of each on PVC solvation. In the same way, *n*-butyl acetate (*δ*_p_ = 3.7) achieved a larger swelling extent than EA (*δ*_p_ = 5.3), although the *δ*_p_ of the latter is much closer to that of PVC (9.2). Remarkably, 1,4-dioxane (*δ*_p_ = 1.8) showed an *R*_swel_ equivalent to that of *n*-butyl acetate, which was ascribed to the fact that the *δ*_h_ of the former solvent (9) significantly exceeds that of the latter (6.3). Thus, one decisively cannot predict solvation by considering only *δ*_p_. The worthy to note that high electronegativity of chlorine makes the neighbouring carbon atom of PVC more prone to nucleophilic attack by electron-donating groups^16^. The polarisation due to chlorine may take into consideration but cannot be justified distinct behaviour of PVC solvation into respective solvents. Therefore, we employed the sum of *δ*_p_ and *δ*_h_ forces (i.e., ‘*δ*t_p,h_’ = (*δ*_p_^2^ + *δ*_h_^2^)^0.5^) as a prediction parameter. For two solvents with similar *δt*_p,h_, such as MiBK and *n*-butyl acetate, one can definitely say that *δ*_p_ has a larger contribution than *δ*_h_. However, abovementioned explanations could not directing such significant order for *δ*_p_ and *δ*_p_ to define the degree of PVC solvation. Here, we can make a general statement that the different proportionating value of *δ*_p_ and *δ*_h_ may show substantial solvation. The solvents which have *δt*_p,h_ in the range of 7.3–12.5, is about closer or similar to the *δt*_p,h_ (11.2) of PVC, showed significant solvation of PVC. Thus, water did not achieve PVC solvation or extraction because of the unfavourable secondary force–induced interactions. At this point, it is worth re-emphasising that polar forces (*δ*_p_, *δ*_h_) are highly directional, while those due to *δ*_d_ are omnidirectional. Conclusively, PVC matrix swelling occur when involved secondary polar forces in PVC-solvent would greatly compatible, whereas chains may stretch (i.e., expansion). This phenomena described as (*δt*_p,h_)_PVC/DINP_ ≈ (*δt*_p,h_ )_solvent_ for polar solvents. Likewise, the shrinkage condition stated as (*δt*_p,h_)_DINP_ ≈ (*δt*_p,h_)_solvent_ ≪ (*δt*_p,h_)_PVC_ for non-polar solvents. Figure 5 shows a general representation of PVC swelling and shrinkage under the influence of organic solvents.

### Plasticiser recovery and solvent regeneration

**Figure 6:**
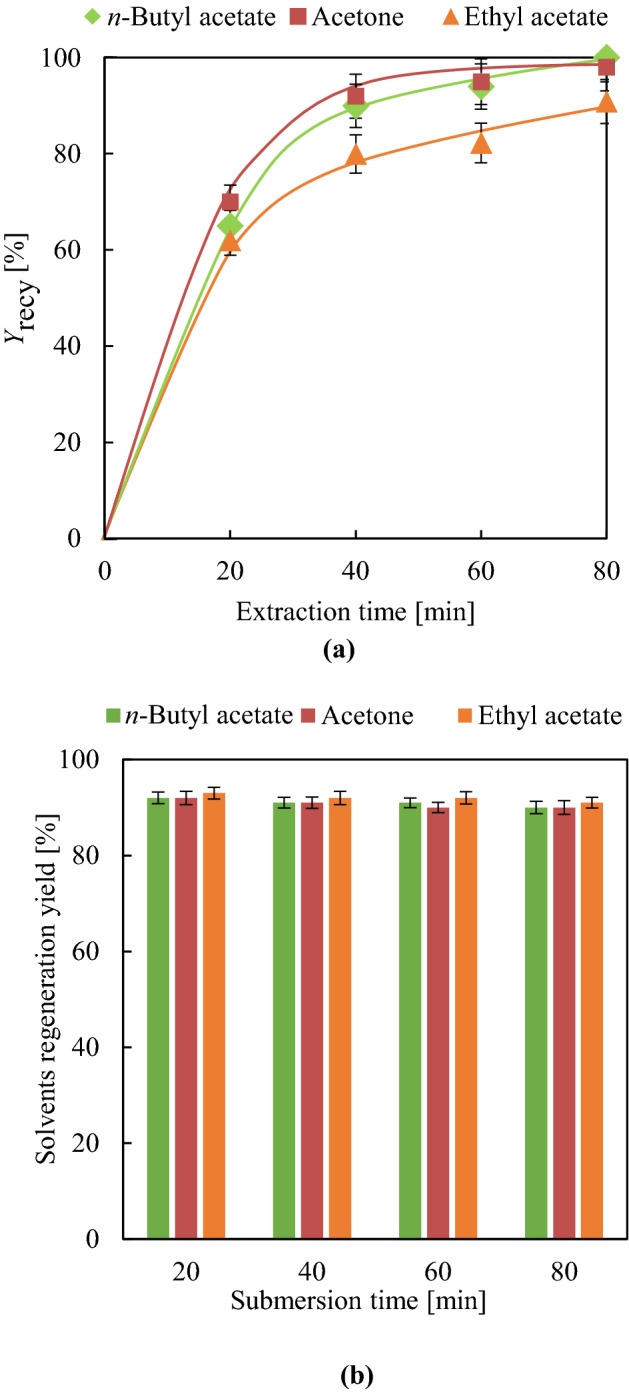
(**a**) Effects of extraction time on DINP *Y*_recy_ [%] and (**b**) effect of submersion time on solvent regeneration yield [%].

DINP solutions obtained after simultaneous swelling and plasticiser extraction were distilled to recover pure DINP and solvents, with detailed distillation conditions provided in the experimental section. The yield of recycled DINP (*Y*_recy_) was determined by ^1^H NMR, with detailed analytical procedures summarised in Supplementary 4. *Y*_recy_ values of 99, 98, and 91% were obtained for acetone, *n*-butyl acetate, and ethyl acetate, respectively, (extraction time = 80 min, Fig. 6a). The ^1^H NMR spectrum of recycled DINP (Supplementary Figs. S6-1 to S6-3) featured only peaks of DINP and residual solvent, indicating that pure DINP could be easily recovered by extraction followed by distillation. At the same time, the solvent (acetone, *n*-butyl acetate, and ethyl acetate) regeneration efficiency of distillation was determined as ~ 90% (Fig. 6b). Thus, DINP recycling and solvent regeneration could be achieved by simple distillation.

### Recovery of Cu and PVC by ball milling of swollen cables

**Figure 7:**
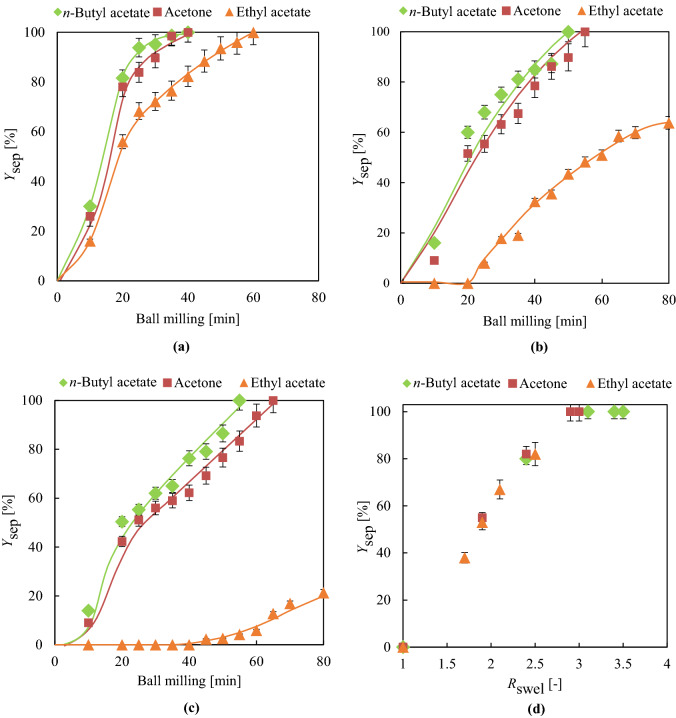
(**a**–**c**) Milling time–dependent *Y*_sep_ [%] for 5-, 10-, and 20-cm cables swollen for 80 min in the designated organic solvent, respectively. (**d**) Relationship between *R*_swel_ and *Y*_sep_ [%] for 40-min ball milling.

Considering the results of swelling and plasticiser extraction experiments, *n*-butyl acetate, acetone, and ethyl acetate were selected for further tests. For dry ball milling tests, 12.8-g swollen cable (5, 10, and 20 cm) samples were prepared by 80-min swelling with shaking, fed into the ball mill reactor containing 20-mm-diameter balls, and crushed at 45 rpm for a maximum time of 80 min. Figures 7a–c show the effects of time on the *Y*_sep_ of 5-, 10-, and 20-cm swollen cables, respectively, showing that in the case of *n*-butyl acetate and acetone, complete separation of 5-cm cables was achieved after 40 min, while in the case of ethyl acetate, complete separation required 60 min. The milling time required for complete separation increased with increasing cable length, e.g., in cases of *n*-butyl acetate and acetone, complete separation of 20-cm cables was achieved after 55 and 65 min, respectively. The high volatility of acetone resulted in quick de-swelling and, hence, in a longer ball milling time. On the other hand, for ethyl acetate, *Y*_sep_ values of 63 and 21% were obtained for 10- and 20-cm cables after 80 min, i.e., complete separation could not be realised. As expected, no separation was observed for untreated cables. Thus, we concluded that *R*_swel_ strongly affects the efficiency of ball milling–induced Cu-PVC separation (Fig. 7d). The successful separation achieved for 20-cm cables is a substantial advance compared to our recent works, in which full separation could be achieved for cables with a maximal length of 3 cm^19,21^.

**Figure 8:**
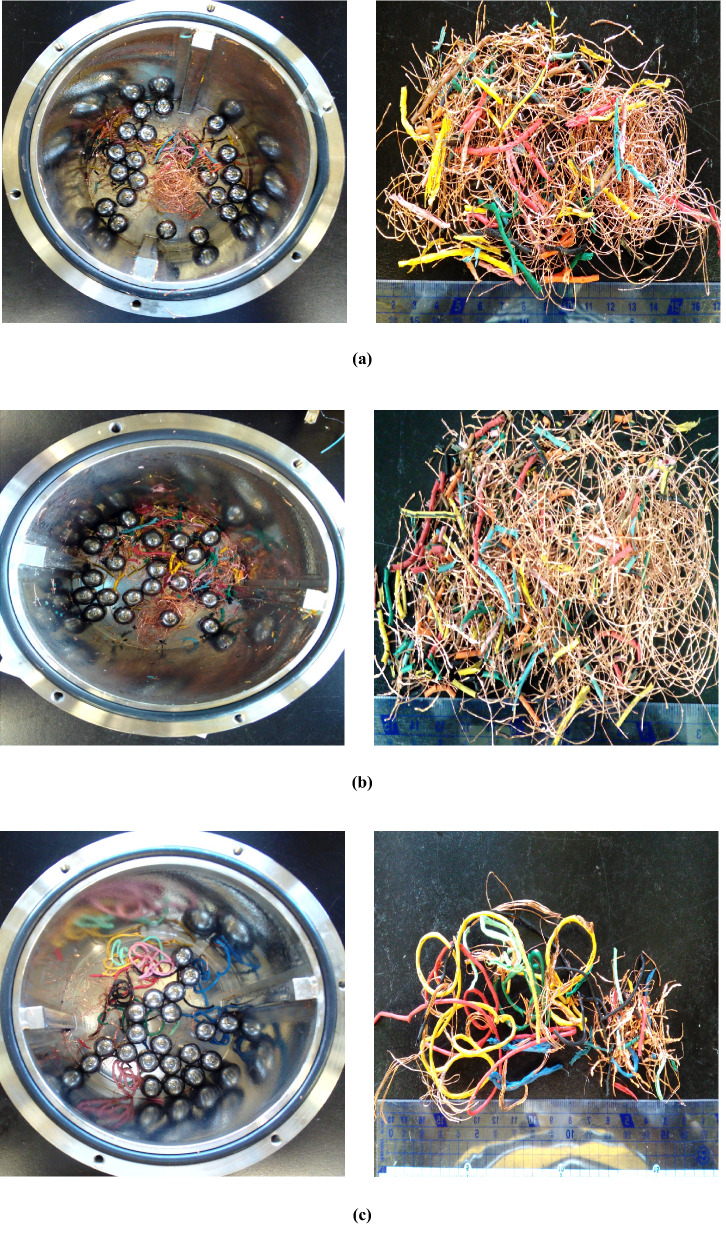
Photographic images of ball milled 20-cm cables before manual sorting. (**a**) *n*-Butyl acetate, (**b**) acetone, (**c**) ethyl acetate.

**Figure 9:**
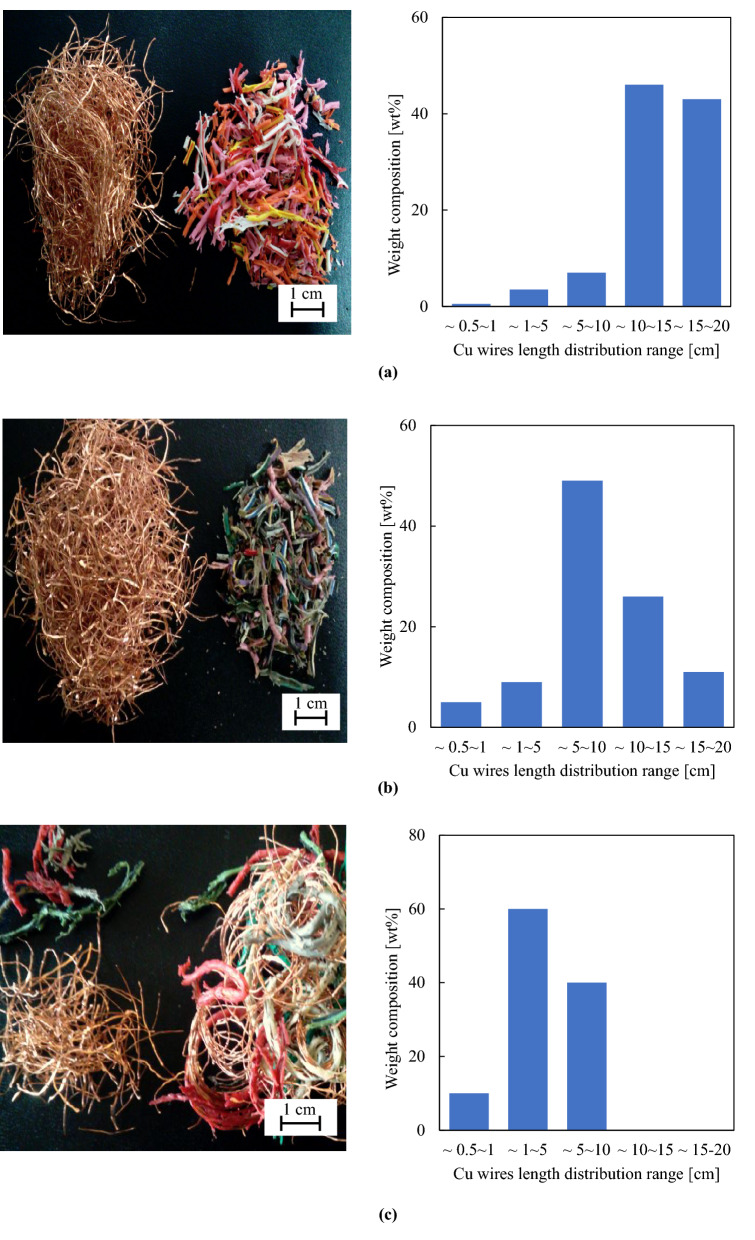
Photographic images of 20-cm cables separated into Cu and PVC after swelling with (**a**) *n*-butyl acetate, (**b**) acetone, (**c**) ethyl acetate. The length distribution of Cu fragments for each solvent is shown in the right-hand images.

The images of ball milled 20-cm cables before and after manual sorting are summarized in Figs. 8 and 9, respectively. The images and Cu wire lengths of 20-cm cables after complete separation show that cables swollen with both *n*-butyl acetate and acetone were completely separated into PVC and Cu (Fig. 9a, b). In the case of *n*-butyl acetate, > 90% of collected Cu wire fragments had a length of ≥ 10 cm, which is a substantial advantage for subsequent Cu refining. For acetone, the recovered Cu fragments were slightly shorter because of the longer milling time required in this case. Figure 9c presents the images and length distribution of Cu fragments recovered from ethyl acetate-treated samples after 80-min ball milling, showing that separation was incomplete and that the obtained Cu wires were much shorter than those obtained from *n*-butyl acetate- and acetone-treated cables because of the longer milling time.

Thus, we successfully developed a novel technique combining PVC swelling with *n*-butyl acetate or acetone and dry ball milling to achieve the quantitative separation of 20-cm-long cables into high-purity PVC coating, Cu wire, and DINP, highlighting the great potential of this method for the recycling of non-uniform, long, and thin cables.

## Materials and methods

### Materials

Waste electric cables were obtained from ELVs (Fig. 1a). Connectors were sorted out, and the cables were cut into 5-, 10- and 20-cm-long pieces. Organic reagents were obtained from Kanto Chemical Co., Inc., (Tokyo, Japan). Coating identity was confirmed by elemental analysis (JM-10/CHN, J-Science Lab Co., Ltd., Japan and YHS-11/S-FClBrI, Science Glove Co., Ltd., Japan) for cables with different diameters and textures (Supplementary Fig. S1). The quantitation of Cu, PVC resin, plasticiser, and THF-insoluble components and plasticiser identification were conducted as described in Supplementaries 2 and 3.

### HSP calculation

The HSP concept considers the action of three general secondary forces between molecules (nonelectrolytes), namely dispersion interaction (*δ*_d_), polar interaction (*δ*_p_), and hydrogen bonding (*δ*_h_) forces. The cohesion energy for PVC-PVC or PVC-DINP separation depends on the participating polar/nonpolar groups and contributing forces. The HSP interaction distances between solvents and PVC (*R*_a_ [S-PVC]) and between solvents and DINP (*R*_a_ [S-DINP]) were calculated and defined as^28^

1 $$R^{{2}}_{{\text{a}}} = {4}\left( {\delta_{{{\text{d}},{\text{s}}}} - \delta_{{{\text{d}},{\text{t}}}} } \right)^{{2}} + {4}\left( {\delta_{{{\text{p}},{\text{s}}}} - \delta_{{{\text{p}},{\text{t}}}} } \right)^{{2}} + {4}\left( {\delta_{{{\text{h}},{\text{s}}}} - \delta_{{{\text{h}},{\text{t}}}} } \right)^{{2}} ,$$

where subscripts ‘s’ and ‘t’ refer to solvent and target material (PVC or DINP), respectively. All HSPs for selected organic solvents, plasticisers, and PVC were calculated using HSPiP software (5th edition, 5.0.06).

### Swelling of PVC coating by submersion into organic solvents

The collected waste cables were cut into 7-cm-long pieces and submerged into organic solvents (100 mL) in sealed 110-mL glass bottles at room temperature for 20, 40, 60, and 80 min. For comparison, submersion tests were also conducted using a water bath shaker (NTS-100V, Tokyo Rikakikai Co, Ltd., Tokyo, Japan) with horizontal shaking motion (150 rpm). For each group of tests, ten cables with different textures (Supplementary Fig. S1) were investigated, and the swelling ratio was reported as the corresponding average. The volume of each cable before and after submersion was measured by an electronic densimeter (MDS-300, Alfa Mirage Co., Ltd., Osaka, Japan) to determine the PVC swelling ratio (*R*_swel_) as

2 $$ R_{{{\text{swel}}}} = V_{{\text{s}}} /V_{0} , $$

where *V*_0_ and *V*_s_ [g/cm^3^] are the cable volumes before and after the swelling test, respectively.

To investigate DINP extraction behaviour during swelling tests, 12.8-g cable samples were submerged into the target solvents (100 mL) in 110-mL sealed glass bottles for 20 or 80 min at ambient temperature. Moreover, cable samples of identical weight were tested with shaking using a water bath shaker for 20, 40, 60, and 80 min at ambient temperature. After the extraction test, the spent solvent was collected into a 250-mL round-bottom flask and evaporated using a rotary evaporator (RE-301-AW, Yamato Scientific Co., Ltd., Tokyo, Japan) at a flask rotation speed of 30 rpm. The water bath temperature was set to 30–60 °C according to solvent volatility. The solvent condenser was cooled to − 5 to − 10 °C by a chiller (CF-300, Yamato Scientific Co., Ltd., Tokyo, Japan) in all experiments. The distillation pressure was controlled using a pressure-controllable vacuum pump (NVP-1000V, Tokyo Rikakikai Co, Ltd., Tokyo, Japan) and was set to 560, 350, 250, 180, 170, 180, or 175 hPa for acetone, THF, ethyl acetate, *n*-propyl formate, isopropyl acetate, benzene, or ethanol, respectively. For high-boiling-point solvents, namely MiBK, *n*-butyl acetate, 1,4-dioxane, *n*-propyl acetate, isobutyl acetate, or water, the vacuum pressure was set to 53, 39, 95, 65, 53, or 73 hPa, respectively. The plasticiser extraction yield (*Y*_ext_ [%]) was determined as

3 $$ Y_{{{\text{ext}}}} = {1}00\% \times {{\left( {w_{{\text{f}}} - w_{{{\text{f}}0}} } \right)} \mathord{\left/ {\vphantom {{\left( {w_{{\text{f}}} - w_{{{\text{f}}0}} } \right)} {W_{{\text{DINP in PVC}}} }}} \right. \kern-\nulldelimiterspace} {W_{{\text{DINP in PVC}}} }}, $$

where *w*_f_ [g] and *w*_f0_ [g] are weights of the flask after distillation and the empty flask, respectively, while *W*_DINP in PVC_ [g] is the amount of DINP in the 12.8-g waste cable sample (see Supplementary 4).

The yields of plasticiser recycled from extraction solvents (*Y*_recy_) were determined by ^1^H NMR (Bruker Advance, 400 MHz, Rheinstetten, Germany) using fumaric acid as an internal standard^21^, with details provided in Supplementary 4.

### Ball milling tests for the recovery of Cu and PVC from swollen cables

After swelling and simultaneous plasticiser recovery, the swollen cables were immediately transferred to the ball mill reactor for PVC separation, as schematically illustrated in Fig. 1c,d. Ball milling tests were carried out in a stainless steel reactor with a length of 160 mm and an inner diameter of 160 mm. For all experiments, 12.8-g cable samples and stainless steel balls with diameters of 15 mm (26 g/ball) or 20 mm (62 g/ball) were used. Rotation speed was controlled by a pot mill rotator (PM-001, AS ONE Co., Tokyo, Japan). The optimal rotation speed, ball size, and ball number were determined as 35 rpm, 20 mm, and 20, respectively (see Supplementary 7). After milling tests, PVC containing organic solvents was completely dried at 60 °C in a vacuum oven overnight. The dried samples were weighed, and the separation yields (*Y*_sep_) were determined as

4 $$ Y_{{{\text{sep}}}} = {1}00\% \times m_{{\text{s}}} /m_{0} , $$

where *m*_0_ and *m*_s_ [g] are the total sample weight after the milling test and the total weight of separated Cu and PVC after the milling test, respectively.

## Supplementary information

Supplementary information
