# Supplementary material for: Simultaneous recovery of high-purity Cu and poly(vinyl chloride) from waste wire harness via swelling followed by ball milling
Source: Sci Rep. 2020 Jul 1;10:10754. doi: 10.1038/s41598-020-67795-9 (PMC7329881; doi:10.1038/s41598-020-67795-9)
Supplement: Supplementary file 1 — Supplementary information [file 41598_2020_67795_MOESM1_ESM.docx]

*Supporting information*

Simultaneous recovery of high-purity Cu and poly(vinyl chloride) from waste wire harness via swelling followed by ball milling

Harendra Kumar, Shogo Kumagai^*^, Tomohito Kameda, Yuko Saito, Toshiaki Yoshioka

Graduate School of Environmental Studies, Tohoku University, 6-6-07 Aoba Aramaki-Aza, Aoba-ku, Sendai, Miyagi 980-8579, Japan

**^*^Corresponding author.** Email: kumagai@tohoku.ac.jp, tel./fax: +81-22-795-7212

**Supplementary 1: Size distribution of the collected cables in the waste harness assembly**

**
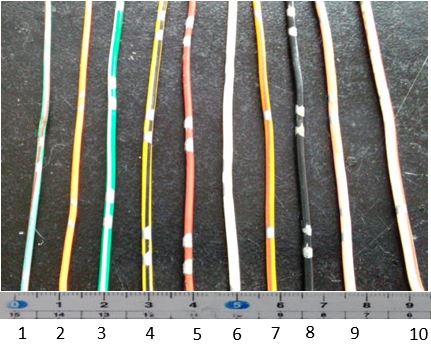
**

Fig. S1. Distribution of cables in the as-received wire harness assembly. Cables with diameters of 1.0 (entries 1 and 2), 1.2 (entries 3–9), and 2.0 mm (entry 10) accounted for 15, 70, and 15% of the total weight, respectively. The Table S1 presents elemental compositions (wt%) of collected cables (entries 1–10).

Table S1. Elemental composition of collected cables

| Cable diameter [mm] | Weight composition [wt%] | | | | |
| --- | --- | --- | --- | --- | --- |
|  | C | H | N | Cl | Balance*^a^* |
| 1.0 | 46.7 | 6.2 | – *^b^* | 35.7 | 11.5 |
| 1.2 | 45.4 | 5.9 | – | 35.3 | 13.4 |
| 2.0 | 45.0 | 5.8 | – | 33.4 | 15.8 |
| *^a^*Mainly oxygen and ash. *^b^*Not detected. | | | | | |

**Supplementary 2: Quantitation of Cu, PVC, and other components in the collected cables**

In the dissolution/precipitation process, waste cables were magnetically stirred for 2 h at 21°C in a glass bottle filled with THF (100 mL). After complete PVC dissolution, Cu was collected and weighed. Insoluble PVC constituents were separated by 20-min centrifugation (H-19F, Kokusan Co., Ltd., Japan) of the solution at 3000 rpm. The supernatant was supplemented with ethanol (100 mL) and sonicated for 2 h to achieve complete PVC precipitation. The precipitated PVC was separated by centrifugation as above and dried in vacuum oven at 60 °C, while the collected solution was distilled using a water bath (50 °C) equipped with a condenser (operated at −5 to −10 °C) to recover the plasticiser and regenerate the solvent. As the recovered plasticiser contained traces of PVC, a further portion of ethanol (50 mL) was added for purification. PVC particles were separated by centrifugation, and the recovered solvent was collected in a fresh flask for renewed distillation. The regenerated PVC, solvent, and plasticiser were finally collected and estimated accordingly.

Fig. S2. Flow chart of cable component characterisation.

**Supplementary 3: Identification of plasticiser** **by ^1^H NMR**

Fig. S3-1. Chemical structure of DINP.

**
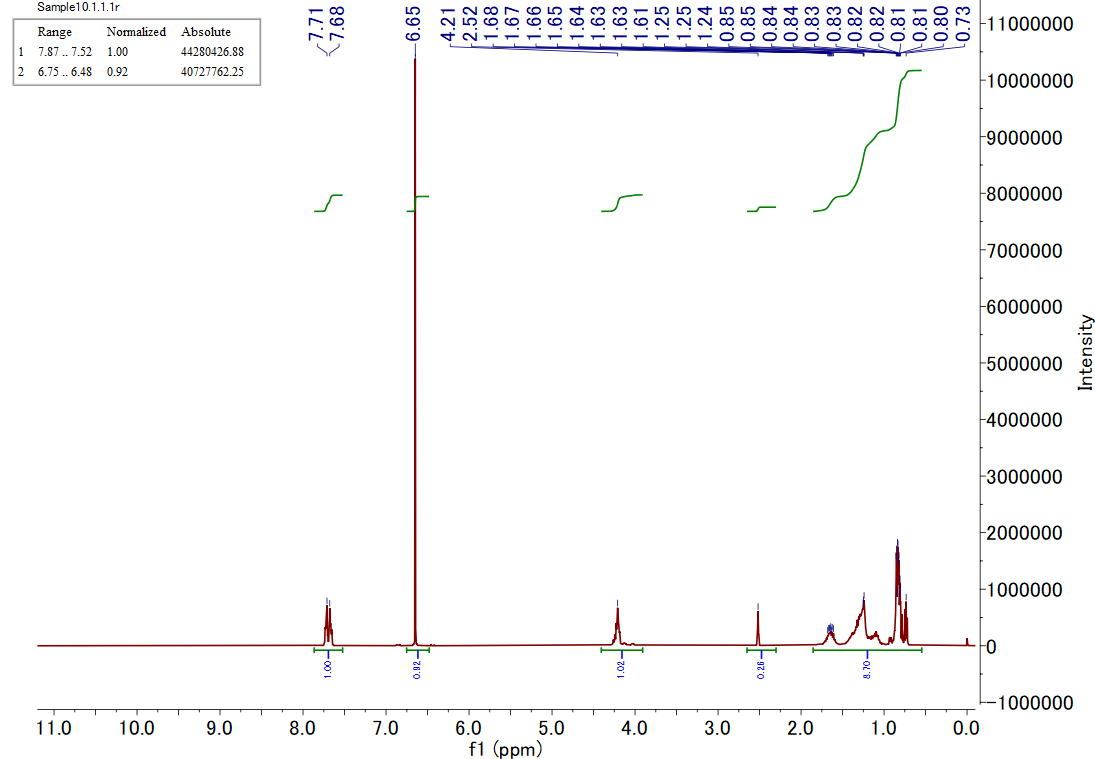
**

Fig S3-2. ^1^H NMR spectrum of pure DINP.

Table S3-1. ^1^H NMR chemical shifts of DINP.

| *δ* (ppm) | Assignment | Coupling constant (*J*) |
| --- | --- | --- |
| 7.71 | H_A_ | 2H, H_1_, dd, ^3^*J*_A–B_ = 5.6 Hz, ^4^*J*_A–B_ = 3.2 Hz |
| 7.68 | H_B_ | 2H, H_2_, dd, ^3^*J*_B–A_ = 5.6 Hz, ^4^*J*_B–A_ = 3.2 Hz |
| 4.20–4.21 | OCH_2_ | (~4H, m) |
| 1.68–0.73 | CH, CH_2_, CH_3_ | (~34H, m). |
| 6.65 | Standard peak (fumaric acid) | |
| 2.51–2.52 | Solvent peak (DMSO) | |

**
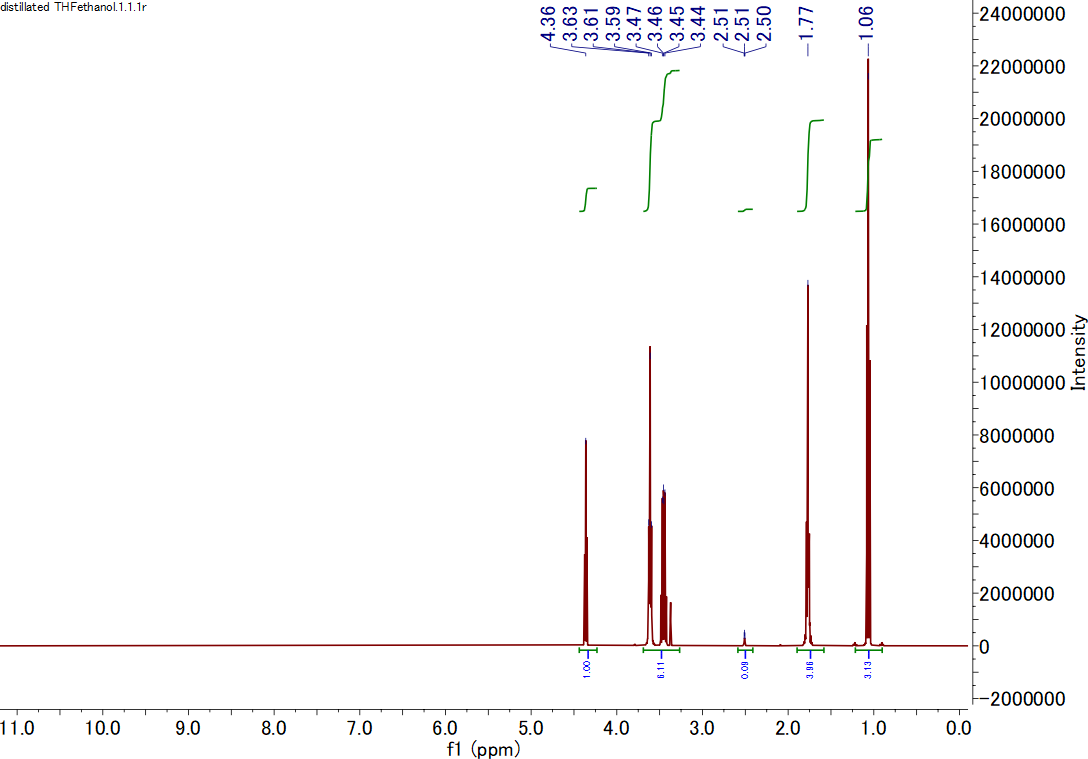
**

Fig S3-3. ^1^H NMR spectrum of distilled solvent (THF and ethanol mixtures) obtained during the cable characterisation process shown in Fig. S2.


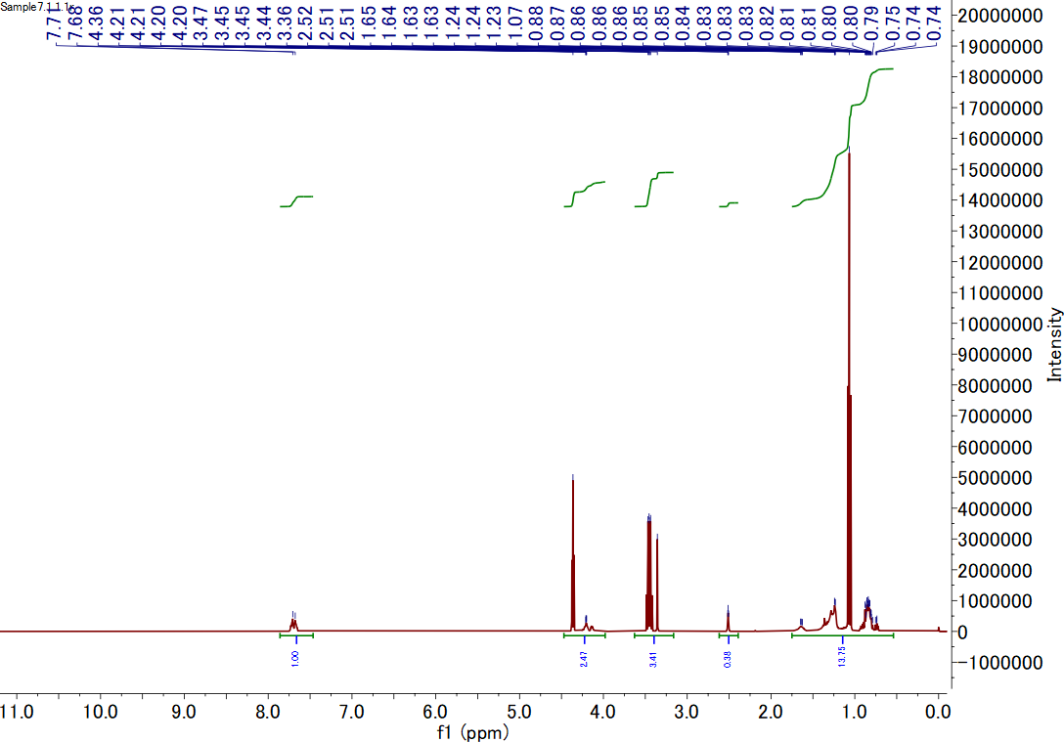


Fig S3-4. ^1^H NMR spectrum of extracted plasticiser obtained after the distillation process shown in Fig. S2.

**
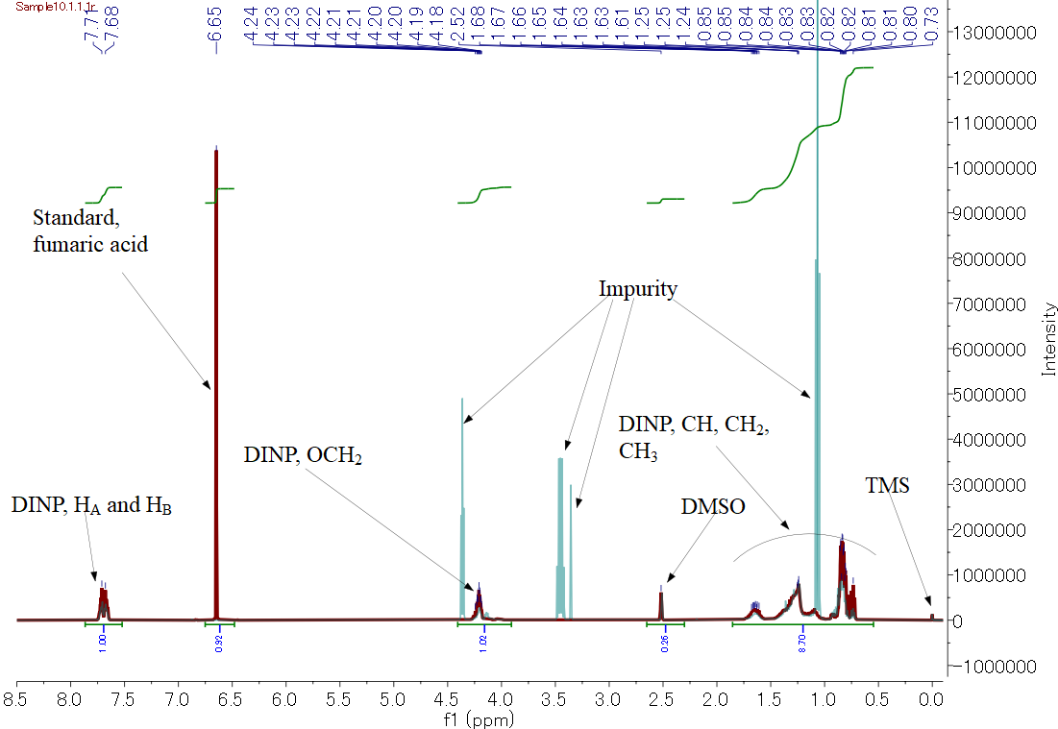
**

Fig S3-5. ^1^H NMR spectrum of pure DINP (red line, Fig. S3-2) superimposed on that of plasticiser extracted from the cable (entry 1) presented in Fig. S1 (blue line). Impurity peaks belong to THF and ethanol (Fig. S3-3). The plasticiser contained in the collected samples (samples 1–10 in Fig. S1) was identified as DINP.

**Supplementary 4: Quantitation of DINP by ^1^H NMR using fumaric acid as an internal standard**

To calculate a coefficient *K* for DINP, a fumaric acid (*m*_F_) – DINP (*m*_DINP_) mixture of known composition was dissolved in DMSO and probed by ^1^H NMR. The area of the fumaric acid peak at 6.65 ppm (*S*_F_) and that of DINP peaks at 7.68 and 7.71 ppm (*S*_DINP_) were determined (Fig. S3-2 and Table S3-1) and used to calculate *K* as

*K* = *m*_DINP_ × *S*_F_ × *M*_F_/(*M*_DINP_ × *S*_DINP_ × *m*_F_), (S1)

where *K* [–] is a coefficient, *M*_DINP_ [g/mol] is the molecular weight of DINP, *m*_DINP_ [mg] is the weight of DINP, *S*_DINP_ [–] is the area of peaks at 7.68 and 7.71 ppm due to the coupling of *ortho*- (H_A_ and H_B_) DINP protons, *M*_F_ [g/mol] is the molecular weight of fumaric acid, *m*_F_ [mg] is the weight of fumaric acid, and *S*_F_ [–] is the area of the fumaric acid peak at 6.65 ppm.

To calculate the recycling yield (*Y*_recy_) of DINP in the 12.8-g waste cable sample, we first calculated the weight of pure DINP (*W*_DINP_) in the NMR sample (which contained a known amount of extracted DINP (*W*_ext_) and a known amount of fumaric acid (*m*_F_)) according to equation (S2). Then, we used the purity factor (*W*_DINP_ divided by *W*_ext_) to calculate the weight of pure recovered DINP (*W*_recy_) in the total DINP (*W*_ext total_) extracted from the 12.8-g waste cable sample as

*W*_DINP_ = *M*_DINP_ × *S*_DINP_ × *m*_F_ × *K*/(*S*_F_ × *M*_F_), (S2)

*W*_recy_ = *W*_DINP_ × *W*_ext total_/*W*_ext_. (S3)

Five replicate experiments (cable sample weight = 12.8-g) were conducted to calculate the average value of *W*_recy_, which, together with the amount of DINP contained in a 12.8-g cable sample (*W*_DINPinPVC_) was used to determine the recycling yield [%] of DINP (*Y*_recy_) as

*Y*_recy_ = 100% × *W*_recy_/*W*_DINP in PVC_. (S4)

**Supplementary 5: Insights into the mechanism of PVC solvation and plasticiser extraction**

Table S5-1 presents the HSPs and properties of PVC resin, DINP and selected organic solvents. The extraction yield (*Y*_ext_ [%]) from PVC cables coating by response of solvent submersion (i.e., with or without shaking) presented in Table S5-2.

Table S5-1. HSPs and properties of PVC resin, DINP, and selected solvents.

| Material/solvent | Density*^a^* [g/mL] | Boiling point [°C] | HSPs*^b^* [MPa^1/2^] | | | |
| --- | --- | --- | --- | --- | --- | --- |
|  |  |  | *δ*_d_ | *δ*_p_ | *δ*_h_ | *^c^δ*t_p,h_ |
| PVC | 1.36-1.4 | *^d^* | 18.8 | 9.2 | 6.3 | 11.2 |
| DINP | 0.98 | 405.7 | 16.6 | 6.6 | 2.9 | 7.2 |
| THF | 0.889 | 66 | 16.8 | 5.7 | 8 | 9.8 |
| Acetone | 0.788 | 56 | 15.5 | 10.4 | 7 | 12.5 |
| Ethyl acetate | 0.897 | 77.1 | 15.8 | 5.3 | 7.2 | 8.9 |
| *n*-Propyl formate | 0.904 | 81 | 15.5 | 7.1 | 8.6 | 11.2 |
| MiBK | 0.801 | 118 | 15.3 | 6.1 | 4.1 | 7.3 |
| *n*-Butyl acetate | 0.883 | 126 | 15.8 | 3.7 | 6.3 | 7.3 |
| 1,4-Dioxane | 1.033 | 101 | 17.5 | 1.8 | 9 | 9.2 |
| *n*-Propyl acetate | 0.888 | 102 | 15.3 | 4.3 | 7.6 | 8.7 |
| Isobutyl acetate | 0.873 | 118 | 15.1 | 3.7 | 6.3 | 7.3 |
| Isopropyl acetate | 0.872 | 89 | 14.9 | 4.5 | 8.2 | 9.4 |
| Benzene | 0.876 | 81.1 | 18.4 | 0 | 2 | 2 |
| Diethyl ether | 0.713 | 34.6 | 14.5 | 2.9 | 5.1 | 5.9 |
| Petroleum ether | 0.64 | 30-60 | 15 | 0 | 0 | 0 |
| Ethanol | 0.789 | 78.4 | 15.8 | 8.8 | 19.4 | 21.3 |
| Water | 0.998 | 100 | 15.6 | 16 | 42.3 | 45.2 |
| *^a^*At 25°C, *^b^*Subscripts ‘d’, ‘p’, and ‘h’ mean dispersion, polar, and hydrogen bonding, respectively, ‘^c^*δ*t_p,h_’=(*δ*_p_^2+*δ*_h_^2)^1⁄2, *^d^*Not reported | | | | | | |

Table S5-2. Extraction yield of DINP (*Y*_ext_) for selected organic solvents and different methods/extraction times.

| Solvent | *Y*_ext_ | | | | | |
| --- | --- | --- | --- | --- | --- | --- |
|  | Without shaking | | With shaking | | | |
|  | 20 min (18 °C) | 80 min (18 °C) | 20 min (21 °C) | 40 min (21 °C) | 60 min  (21 °C) | 80 min  (21 °C) |
| THF | Dissolution | | | | | |
| Acetone | 37.4 ± 1.4*^a^* | 59.4 ± 1.9 | 70.2 ± 3.6 | 94.1 ± 3.1 | 95.6 ± 3 | 98.7 ± 2.0 |
| EA | 33.3 ± 1.7 | 41.9 ± 1.4 | 63.6 ± 3.3 | 81.3 ± 2.9 | 85.8 ± 2.8 | 90.9 ± 2.3 |
| *n*-Propyl formate | 40.9 ± 2.8 | 51.0 ± 2.1 | 66.2 ± 3.3 | 83.3 ± 1.8 | 95.5 ± 1.9 | 99.0 ± 2.2 |
| MiBK | Partial dissolution | | | | | |
| *n*-Butyl acetate | 33.8 ± 3.8 | 57.3 ± 1.9 | 66.3 ± 3.8 | 94.9 ± 3.4 | 97.7 ± 3.3 | 97.2 ± 2.9 |
| 1,4-Dioxane | 38.4 ± 2.2 | 48.5 ± 3.1 | 69.4 ± 1.9 | 79.5 ± 3.6 | 88.9 ± 2.3 | 96.5 ± 2.1 |
| *n*-Propyl acetate | 32.3 ± 3.6 | 49.0 ± 4.3 | 71.7 ± 2.2 | 78.5 ± 2.5 | 85.4 ± 2.0 | 95.4 ± 1.1 |
| Isobutyl acetate | 40.7 ± 2.3 | 44.2 ± 2.2 | 70.2 ± 1.8 | 78.80 ± 2.4 | 93.2 ± 2.3 | 94.7 ± 1.9 |
| Isopropyl acetate | 30.6 ± 2.3 | 41.2 ± 1.7 | 63.7 ± 1.9 | 76.28 ± 3.5 | 82.8 ± 1.8 | 97.8 ± 1.7 |
| Benzene | 18.4 ± 4.3 | 25.3 ± 2.9 | 30.3 ± 1.1 | 38.4 ± 3.7 | 55.1 ± 2.1 | 80.3 ± 1.1 |
| Diethyl ether | 26.5 ± 3.6 | 33.6 ± 2.7 | 41.2 ± 2.3 | 76.2 ± 5.2 | 84.3 ± 3.4 | 97.2 ± 2.1 |
| Petroleum ether | 10.6 ± 0.8 | 16.2 ± 1.2 | 18.4 ± 0.1 | 29.81 ± 2.1 | 44.2 ± 3.0 | 47.9 ± 1.2 |
| Ethanol | 0.5 ± 0.0 | 1.3 ± 0.1 | 1.5 ± 0.0 | 2.53 ± 0.0 | 3.0 ± 0.0 | 4.5 ± 0.0 |
| Water | 0 | 0 | 0 | 0 | 0 | 0 |
| *^a^*Standard deviation determined from two experiments | | | | | | |

**Supplementary 6: Recovery of DINP by distillation**

**
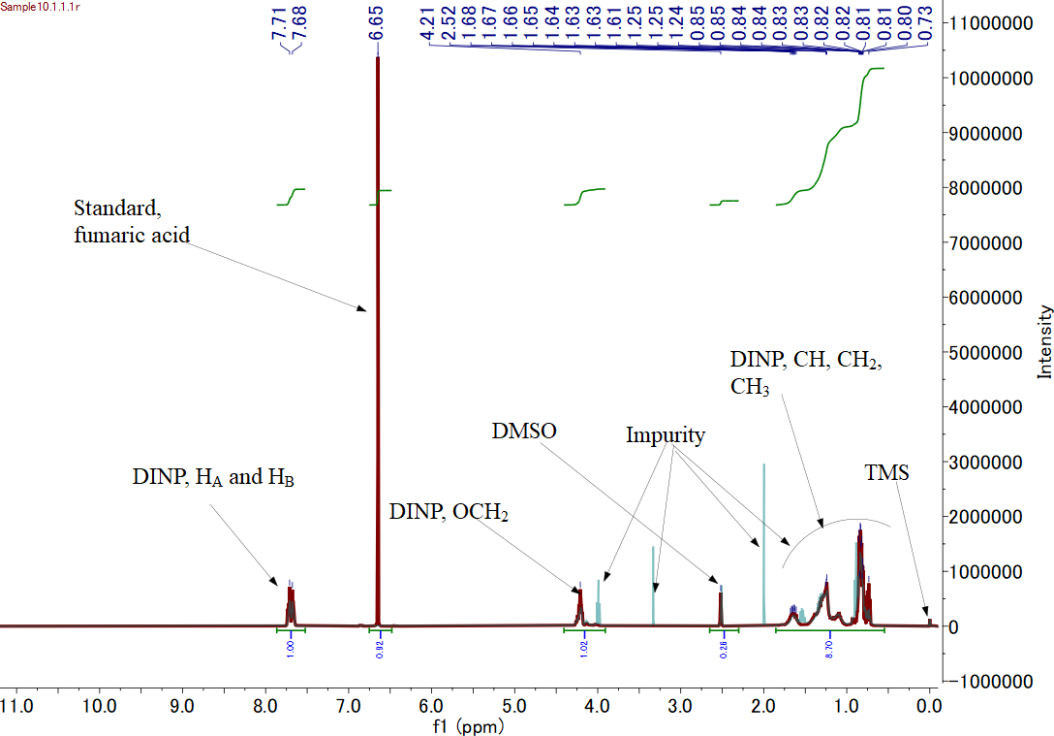
**

Fig. S6-1. ^1^H NMR spectrum of pure DINP (red line, Fig. S3-3) superimposed onto that of DINP recycled from *n*-butyl acetate collected after 80-min extraction (blue line). Impurity peaks belong to *n*-butyl acetate.

**
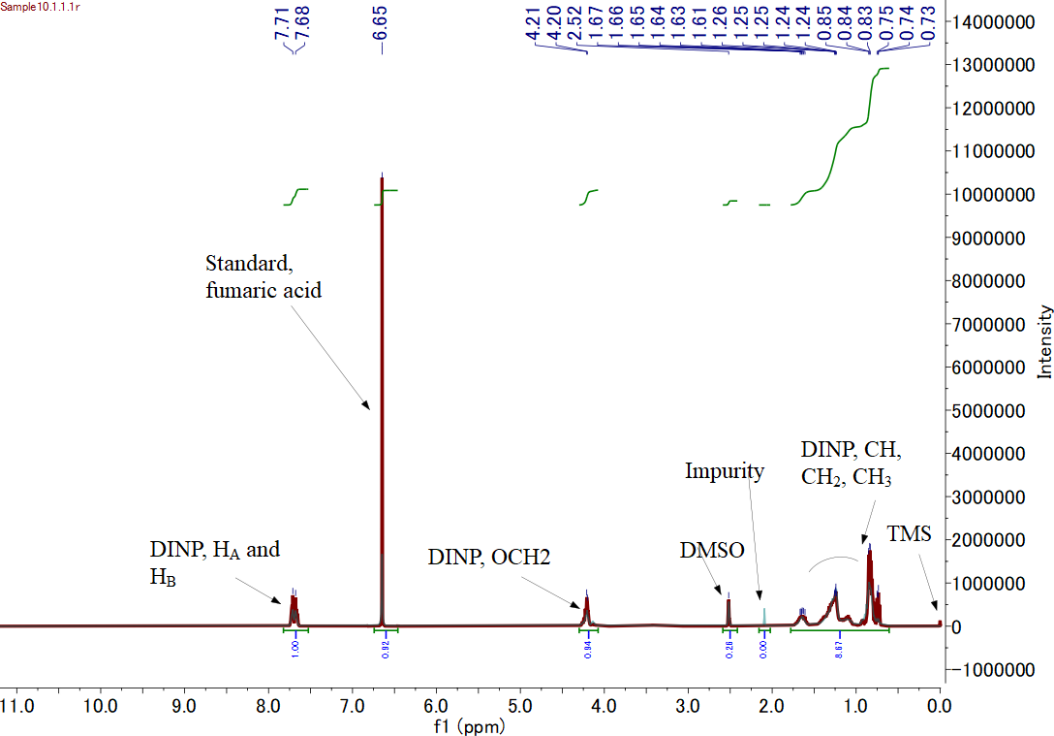
**

Fig. S6-2. ^1^H NMR spectrum of pure DINP (red line, Fig. S3-3) superimposed onto that of DINP recycled from acetone collected after 80-min extraction (blue line). Impurity peaks belong to acetone.

**
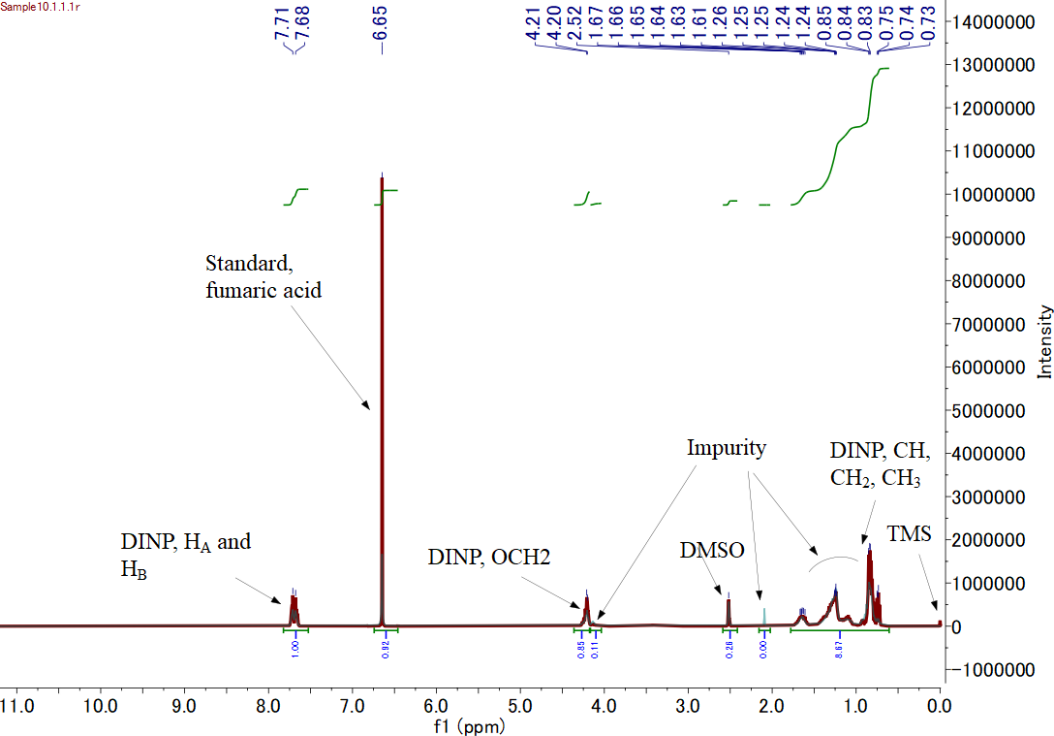
**

Fig. S6-3. ^1^H NMR spectrum of pure DINP (red line, Fig. S3-3) superimposed onto that of DINP recycled from EA collected after 80-min extraction (blue line). Impurity peaks belong to EA.

**Supplementary 7: Influences of rotation speed, ball size, and cable length on separation efficiency**

To investigate the effects of ball size on the separation of swollen cables (5-, 10-, and 20-cm-long), ball milling tests were conducted with 15- and 20-mm-diameter balls. Typically, a 12.8-g sample swollen with *n*-butyl acetate for 80 min and 20 balls were loaded into the reactor. The rotation speed and milling time were set to 35 rpm and 40 min, respectively. The effect of cable length on separation yield is summarised in Fig. S7-1, which reveals that 5-cm cables were almost completely separated in cases of both 15- and 20-mm-diameter balls. The separation yield drastically decreased with increasing cable length when 15-mm-diameter balls were employed but was maintained in the case of 20-mm-diameter balls. Therefore, we concluded that 20-mm-diameter balls provided sufficient impact energy for the separation of wire harness cables and were suitable for testing the effects of different pre-treatments.

Reactor rotation speed is also an important factor influencing separation efficiency. The effects of rotation speed on separation yield were investigated using the 5-cm-length sample employed in Fig. S7-1, with the results summarised in Fig. S7-2. At a rotation speed of 10 rpm, the impact energy was insufficiently high, which resulted in a very low separation yield. On the other hand, the separation yield at 45 rpm was higher than that at 35 rpm, while yielded Cu wires were broken into shorter length. The rotation speed of 35 rpm provided impact energy sufficient for the separation of wire harness cables, while the speed of 53 rpm was excessively high.

Fig. S7-1. Effects of ball size on the separation yield of variable-length cables swollen with *n*-butyl acetate.

Fig. S7-2. Effects of rotation speed on the separation yield of 5-cm cables swollen with *n*-butyl acetate for 20 min.
